# Progress and projections in the program to eliminate trachoma

**DOI:** 10.1371/journal.pntd.0005402

**Published:** 2017-04-20

**Authors:** Paul M. Emerson, Pamela J. Hooper, Virginia Sarah

**Affiliations:** 1 International Trachoma Initiative, Decatur, Georgia, United States of America; 2 International Coalition for Trachoma Control, London, United Kingdom; Baylor College of Medicine, Texas Children's Hospital, UNITED STATES

## The problem of trachoma

Trachoma is a progressive and miserable disease. Initiated by frequent and repeated ocular infections with the bacterium *Chlamydia trachomatis*, scars on the inside of the eyelids contract, disrupting the lid margin and causing the eyelashes to rotate inwards until they rest against the eye. With each excruciating blink, the lashes damage the sensitive cornea leading to corneal opacity and irreversible blindness.

Trachoma has been with us for a long time; it is among the conditions catalogued in the oldest medical text—the Ebers Papyrus. Taking into account life expectancies too short to allow many people to develop cataracts, trachoma was likely the leading cause of blindness before the Industrial Revolution and the consequent increase in life expectancy.

In our view, the integrated and holistic SAFE strategy to eliminate trachoma—a strategy based on surgical correction of misplaced lashes, the mass distribution of donated antibiotics, and the promotion of water, sanitation, and hygiene (WASH)—makes the program the most compelling of the neglected tropical disease (NTD) elimination programs. SAFE implementation goes far beyond “just” putting trachoma in the crosshairs for elimination as a public problem. Whilst we are used to the concept of collateral damage, the SAFE strategy comes with considerable collateral benefits. Such benefits include the improved quality of life associated with surgery, particularly for patients who are yet to suffer loss of vision [1]. An annual dose of azithromycin increases child survivorship by reducing mortality from common ailments like malaria, bacterial respiratory tract infections, and diarrhea, the three of which remain the primary killers of children living in poverty—children who do not have routine access to antibiotics [2, 3]. Freedom from trachoma does not have to wait for development. Development is the result of freedom from trachoma.

## Scaling up the donation program

**Fig 1:**
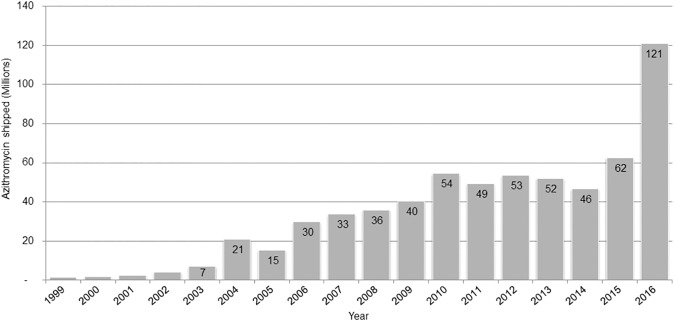
Total doses of Zithromax shipped to trachoma-endemic countries by year (updated 31 December, 2016).

When the World Health Assembly adopted the elimination of trachoma in 1998, the geographical distribution of the disease and numbers affected could only be estimated. Without a clear understanding of the scale of the problem, the pharmaceutical company Pfizer Inc. agreed to an initial donation of 10 million doses of its antibiotic azithromycin through the International Trachoma Initiative as a proof of concept after it was demonstrated that a single oral dose was as effective as the current standard treatment of six weeks of topical ophthalmic tetracycline [4]. The first countries to receive donations, including Ghana, Morocco, Nepal, Tanzania, and Vietnam, demonstrated that the donated azithromycin could be managed without mixing it with the general pharmaceutical supply and developed best practices in managing the mass drug administration (MDA). As a result, the donation was quickly increased to 35 million doses. With the incorporation of the International Trachoma Initiative into The Task Force for Global Health in 2009, the donation program moved into a new period of scale-up, serving an average 15 countries with approximately 45 million doses of azithromycin annually. Between 1998 and 2012, just over 1,000 health districts had conducted rigorous epidemiological surveys to assess the prevalence of trachoma. Then came a game changer in trachoma elimination efforts: the Global Trachoma Mapping Project (GTMP). Through the extremely successful implementation of GTMP, over 1,500 additional health districts were surveyed between 2012 and 2016 [5]. With the global prevalence map just about complete, and all the data freely available and updated regularly via the online map (www.trachomaatlas.org), the scale of the task ahead of us could be visualized. The target of eliminating trachoma set in 1998 suddenly felt real and achievable. Now we were not shooting in the dark, new financial resources were made available (largely from the United States and United Kingdom governments and the Queen Elizabeth Diamond Jubilee Trust), and Pfizer Inc. once again doubled the size of its donation to over 100 million doses a year in 2016 (Fig 1). That same year, six implementing organizations (The Carter Center, the Fred Hollows Foundation, Helen Keller International, Orbis, RTI, and Sightsavers) were each scheduled to support the distribution of over 10 million doses within the context of the full SAFE strategy. By 2016, it was clear that the SAFE strategy worked and the global program was fully at scale.

## Getting to elimination: Scaling down the donation program

Endemic districts are considered to warrant MDA with azithromycin until such time as the clinical signs of active disease in children aged one to nine years (TF_1-9_) fall below 5% and stay below 5% for two years. In calendar years 2014 and 2015, 143 health districts with a total population of 29.7 million people reached this elimination target. In addition, a new country-level validation process has been developed, and Morocco joins Oman in being validated as having eliminated trachoma as a public health problem by the World Health Organization [6]. Of the original countries in the donation program, four are likely to have crossed the finish line.

The trachoma community is now working to finalize a forecast model, based on conservative assumptions, that will allow us to determine when the global elimination of trachoma will be achieved. Because a country cannot declare elimination of trachoma until the very last of its endemic districts is demonstrated to reach and remain below 5% TF_1-9_, countries will be validated relatively slowly. However, this masks the remarkable progress being made in hundreds of districts around the world. By the year 2020, we estimate that at least 70% of the endemic districts will have reached the transmission target of TF_1-9_ below 5%. This forecast will allow the global program to identify areas where an infusion of additional funding, an alternative treatment strategy, or other creative approaches will be required in order to speed the achievement of our elimination goals.

## The next 15 years—Maintaining the momentum

The astonishing progress shown in the global program demonstrates that trachoma can be eliminated as a public health problem. The targets can be reached. People need no longer live with the prospect of having their corneas scratched out by their own eyelashes. The global trachoma program is public health at its best. The allure of “getting to zero” and freeing humankind from a disease that has plagued it for millennia is inspiring, but considerable challenges remain [7].

Some districts, most notably in northern Ethiopia and central Tanzania, are responding to intervention slower than the rest of the world: ten or more years of intervention with many rounds of MDA are often insufficient to reach the elimination target [8]. Alternative, more aggressive treatment strategies may be needed.

For trachoma to be eliminated, every endemic community must be reached. The most marginalized people—indigenous tribes in the Amazon forest and hunter-gatherers in Africa, refugees and internally displaced people, and those living in areas of armed conflict—must be reached, in addition to the settled populations. Sadly, the most marginalized people and those living in war-torn areas are often those most at risk of neglected tropical diseases.

The evidence base for the global program has been developed and refined largely on the backs of ongoing national programs: we have been building the ship as we sailed it [9]. Adequate investment in primary research is absolutely essential, and funding for trachoma research to date has not been proportional to the problem. As we get closer to the end, those places in which we have failed to fully understand the transmission dynamics may remain as islands of potential infection, preventing achievement of global elimination. We must learn lessons from other disease elimination or eradication efforts; for example, the unexpected discovery of dogs with Guinea worm disease, suggesting the existence of an unknown paratenic host for the parasite, only came to light after 99.9% of the global burden of Guinea worm disease was eliminated [10].

The global program has made tremendous progress. We have come further than we could possibly have hoped at the launch in 1998; but as strong as the global program is, we are also fragile. Any significant change in philanthropic philosophy, power, and politics can trump public health gains. The global program remains dependent on strong political will to succeed in endemic countries, the active participation of over 200 million people at risk, an unfaltering supply of donated medicine, a continuous pipeline of donor funds, increasing support to operational and basic science research, and access to all endemic areas. Despite the challenges, it is possible to eliminate trachoma as a public health problem. The world deserves our continued efforts and best work now so that future generations will never know the pain and suffering of trachoma.

## Funding statement

No funding was provided for the preparation of this opinion piece. This opinion piece was prepared by PME, PJH and VS in their personal capacity. The views and opinions expressed are the views and opinions of the authors themselves and do not reflect the opinions, views or policy of the Task Force for Global Health, Pfizer Inc. or the International Coalition for Trachoma Control.
